# Bilateral asymmetrical herpes-zoster with Ramsay hunt syndrome in an immunocompetent adult

**DOI:** 10.1186/s12985-020-01392-0

**Published:** 2020-08-15

**Authors:** Siqi Dai, Xiaowen Huang, Yuxiang Chen, Menglei Wang, Huanxin Zheng, Kang Zeng, Li Li

**Affiliations:** Department of Dermatology and Venereology, Nanfang Hospital, Southern Medical University, No. 1838 North Guangzhou Avenue, Guangzhou, 510515 Guangdong China

**Keywords:** Antiviral therapy, Bilateral herpes zoster (BHZ), Glucocorticoid, Ramsay hunt syndrome (RHS), Varicella-zoster virus (VZV)

## Abstract

**Background:**

Bilateral herpes zoster (BHZ) is an atypical presentation of herpes zoster (HZ), with few cases reported before. Ramsay Hunt syndrome (RHS) is an uncommon complication of VZV infection. Cases of BHZ with RHS in immunocompetent adults have been reported rarely.

**Case presentation:**

We described an immunocompetent adult who suffered from left-sided thoracic herpes zoster and contralateral RHS simultaneously, and summarizes the characteristics of BHZ.

**Conclusions:**

Cases of BHZ with RHS in immunocompetent adults have not been reported previously. Antivirus - glucocorticoid combination therapy showed a good effect in this case.

## Introduction

Herpes zoster (HZ) is a common infection caused by the varicella-zoster virus (VZV), usually happened in patients in hypoimmunity. VZV remains dormant in nerve tissue until activated. And then it can move along the nerve fibers, lurking in the posterior root ganglion of the spinal cord. Patients with HZ present as erythema, pinhead-sized blister, exudate, neuralgia, which usually do not cross the midline of the body [1]. When bilateral dermatomes are involved, called bilateral herpes zoster (BHZ), Ramsay Hunt syndrome (RHS) is an infrequent, severe presentation of VZV reactivation in the geniculate ganglion. Patients with RHS often appear as herpes of external auditory meatus or tympanic membrane, earache, and facial numbness because of viral invasions to the facial nerve and auditory nerve [2]. Herein, we report an immunocompetent adult suffered from BHZ and RHS simultaneously. As far as we know, there are no other known cases like this patient.

## Case report

**Fig. 1:**
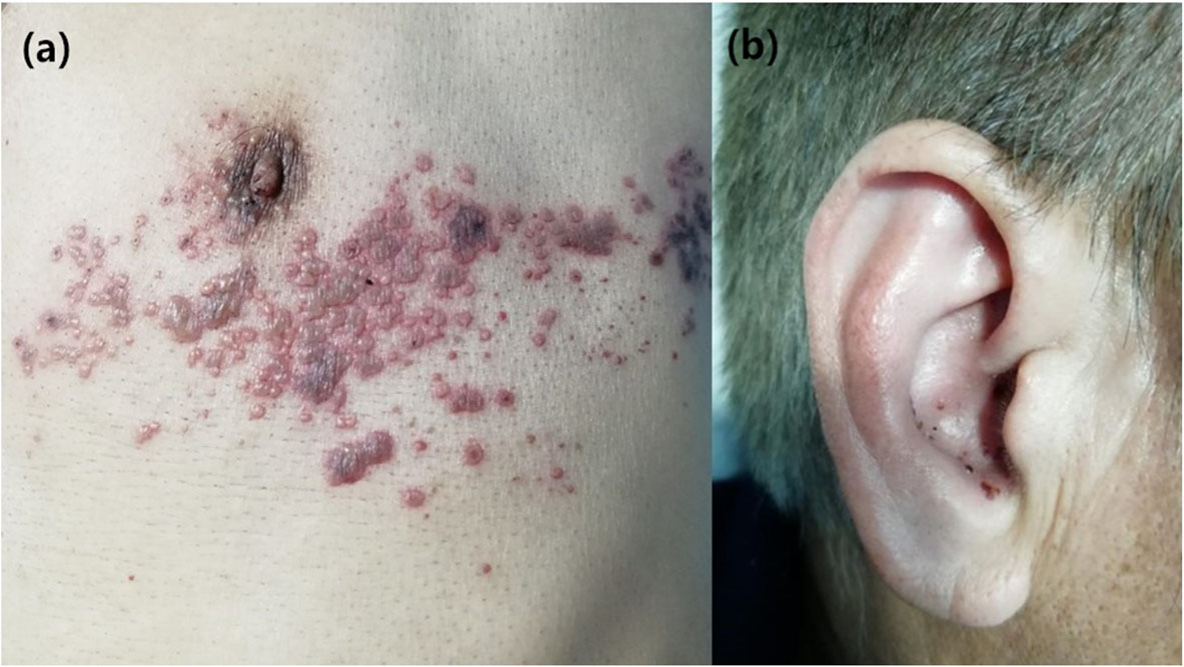
Diffused erythema and clustered vesicles affecting the left T4-T5 dermatome, shown in panel (**a**). Facial nerve and auditory nerve was involved in zoster of the right ear, shown in panel (**b**)

A 55 year-old-male presented to the dermatology clinic with diffused erythema and clustered vesicles affecting the left chest and right ear (Fig. 1a, b). He complained severe pain in the affected region. One week before, some vesicles appeared after taking alcohol. The typically neuropathic pain, such as burning sensation, Shock-like pain, stabbing pain, and feeling of numbness, has been accompanied by the rash. He took some anodyne in an attempt to relieve the pain, but it does not affect reducing symptoms. In the following days, there was a facial asymmetry that occurred in this patient, and the patient developed exudating in his right ear canal.

In the physical examination, grouped blisters, even hernorrhagicbulls with an erythematous base, appeared on his left chest and back along T4-T6 dermatomes. Some blisters had been ruptured and scabbed. The patient’s face was asymmetrical with the droopy corner of the right mouth, and his right nasolabial fold became flattened, and the right eyelid could not be completely closed. The tympanic membrane was integral, but yellow to white exudation was observed on the surface of the external auditory canal.

**Fig. 2:**
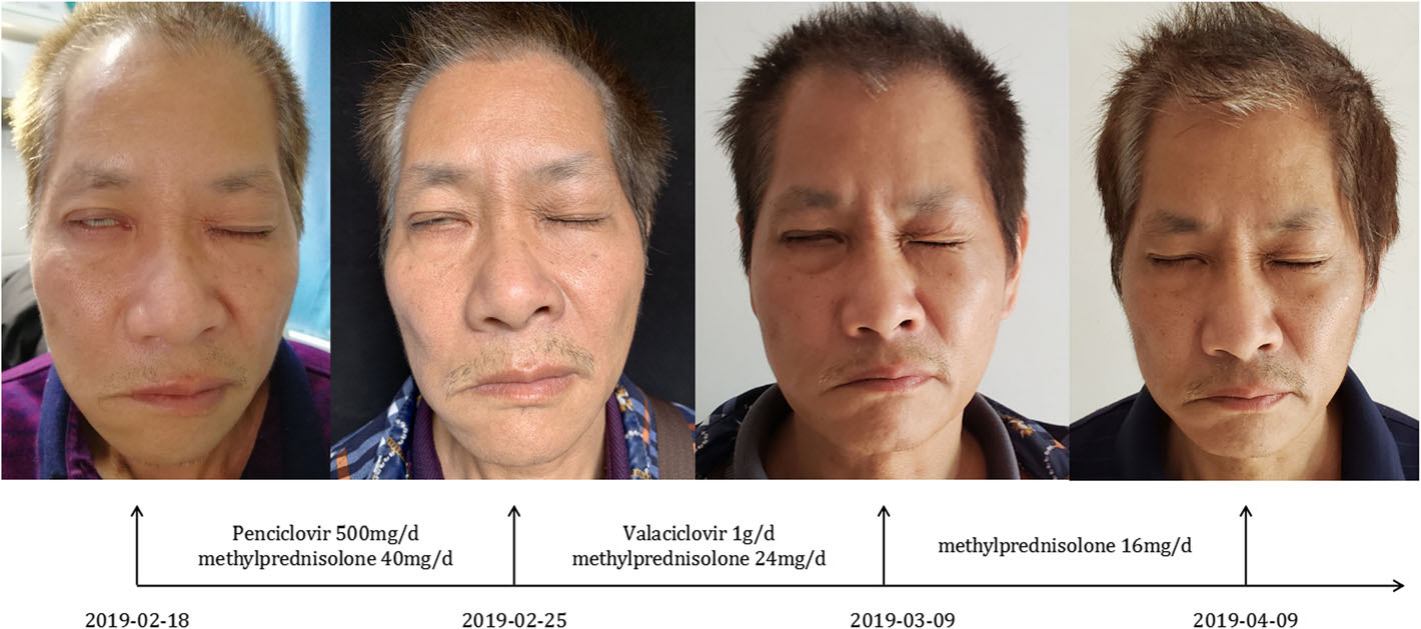
Some noteworthy information with regards to the clinical effect has been recorded within the observation of this patient during the period from Feb 18 to Apr 09 in 2019. From Feb 18 to Feb 25, the right facial palsy with lagophthalmus had initially improved with a slight change. By Mar 9, the patient could almost close his right eyelid. However, there was no significant change for flattened nasolabial old and droopy corners of the mouth on the right side within this period. By Apr 9, the right facial palsy had been resolved

Laboratory investigations, pure tone audiometry tests, and ear examination were routine. He did not have any chronic disease, medical history, recent weight loss, or exposure to any infectious diseases. The patient was not performed virology tests because the disease could be diagnosed based on typical clinical manifestations. He received the treatment of penciclovir 250 mg twice by dripping and methylprednisolone 40 mg once daily. Pain relief with oral gabapentin and super laser irradiation. By using this therapy for 1 week, vesicles over the right ear and left chest had been absorbed and crusted. The patient could perceive the pain relieving effect. Besides, the right facial palsy with lagophthalmus had slightly improved during this period. Continuation of hospitalization has been advised to the patient, but he denied and left the hospital voluntarily. After being discharged, he took oral valaciclovir 500 mg twice daily, methylprednisolone 24 mg per day, and mecobalamin for 7 days. Besides, acupuncture therapy was conducted on the patient once a week in a traditional Chinese medicine hospital. Two weeks after being discharged, the patient could almost close his right eyelid, and his feeling of pain was entirely resolved. And then, the dose of methylprednisolone had been reduced gradually and discontinued within 1 month. After 2 months follow-up, this patient could close his right eyelid completely, flattened nasolabial fold, and droopy corner of the mouth on his right side has also been improved. (Fig. 2).

## Discussion

Herpes Zoster is a viral disease caused by VZV, characterized as unilateral erythema, blisters, and pain. It usually affected one limited side of the body. When bilateral dermatomes are involved, called bilateral herpes zoster (BHZ), which is an atypical presentation of HZ, although it has an incidence rate under 0.1% and is usually found in immunosuppressed or senile patients [3], it also happened when VZV escapes unexpectedly from cellular immunity in healthy people. We have reviewed literature and found 40 cases of BHZ have been reported, as illustrated in Table 1. (References can be found in the Supplement 1), The data of those cases was organized with attributes of age, gender, involving dermatomes, underlying diseases, and treatments. It showed that the age of patients with BHZ ranged widely from 3 to 91, with an average of 43.35. Among them, 25 are males. Besides, the thoracic dermatome was mostly involved in BHZ, which was consistent with the previous reports [4]. Furthermore, 21 of the patients were immunocompetent, which includinten10 symmetrical and 11 asymmetrical lesions. The remaining 19 patients were immunocompromised, 7 of them have cancer, and 2 of them have acquired immune deficiency syndrome (AIDS) as underlying diseases. For most patients, the symptoms were relieved after the treatment of acyclovir or famciclovir.

**Table 1:** Overview of reported cases of bilateral herpes zoster (BHZ)

NO.	Age (years)	Sex	Dermatomes	Symmetry	Underlying disease	Treatment	Reference (see supplementary materials for details)
1	61	M	R: T2–3 L: C5-T1	Asymmetry	/	VCV oral 1 g/q8h 7 days	1
2	24	F	R: L1–2 L: maxillary dermatome	Asymmetry	/	ACV oral 800 mg × 5/d 10 days	2
3	7	M	R: C4 L: T3–4, L2	Asymmetry	/	ACV oral 800 mg × 5/d 7 days	3
4	16	M	R: trigeminal nerve dermatome L: T4–7	Asymmetry	/	ACV oral 500 mg × 3/d 7 days	4
5	45	M	R: T9 L: trigeminal nerve dermatome	Asymmetry	/	ACV IV 10 mg/kg × 3/d	5
6	26	M	R: T8 L: T9	Asymmetry	/	ACV 800 mg × 5/d 7 days	6
7	73	F	R: L1–2 L: T9–10	Asymmetry	/	Isoprinosine 1.000 mg × 4/d	7
8	60	M	R: trigeminal nerve dermatome, forearm L: back	Asymmetry	/	Prednisolone oral 40 mg/d topical ACV and steroids	8
9	40	F	R: neck and ear L: neck and shoulder	Asymmetry	/	Quinine, iron and sulphateof magnesia oral	9
10	28	M	R: T8–9 L: T12, L1–2	Asymmetry	/	ACV oral 800 mg × 5/d 7 days	10
11	14	M	R: forehead L: L1	Asymmetry	/	ACV IV 1500 mg/m^2^/dClindamycin IV	11
12	21	M	Trigeminal nerve dermatome	Symmetry	/	?	12
13	3	M	Face, nose, chin and ear	Symmetry	/	Triple sulfa and penicillin	12
14	41	F	Neck	Symmetry	/	No treatment	12
15	15	M	T7–9	Symmetry	/	ACV 800 mg × 5/d 7 days	13
16	33	F	Upper sacral areas, hips, and upper part of the buttocks bilaterally	Symmetry	/	?	14
17	24	M	Chest	Symmetry	/	?	15
18	18	M	Face and head	Symmetry	/	?	16
19	54	M	Neck	Symmetry	/	ACV IV 21 days	17
20	23	M	Forehead and temporal areas	Symmetry	/	?	18
21	55	M	T4	Symmetry	/	ACV 800 mg × 5/d 7 days	10
22	75	M	Trigeminal nerve dermatome	Symmetry	Prostate carcinoma	ACV	19
23	70	M	R: C4, T2 L: L1–2	Asymmetry	CLL	ACV IV 10 mg/kg/8 h	20
24	70	M	R: C4, T4 L: T9–10	Asymmetry	Diabetic, CKD and MM	ACV 375 mg/d 10 days	21
25	39	F	T8	Symmetry	After thoracoscopic splanchnicectomy	ACV oral 800 mg × 5/d 5 days	22
26	31	M	Eyes	Symmetry	AIDS	ACV oral 800 mg × 5/d	23
27	66	F	R: C4–5 L: facial and the posterior auricular nerves	Asymmetry	Rheumatism and heart disease	?	24
28	63	M	T11	Symmetry	ESRD	VCV oral 250 mg/d	25
29	54	F	R: T5–7 L: T10	Asymmetry	MM	FCV	26
30	52	F	Face and neck	Symmetry	SLE, TB	?	27
31	21	M	R: T9–10 L: T9	Asymmetry	UC	Antiviral IV	28
32	47	F	L4–5, S1	Symmetry	Renal transplantation	VCV oral 1 g tid 7 daysVCV oral 1 g/d 6 months	29
33	27	M	R: T9 L: T6–8	Asymmetry	Pharyngotonsillitis	Oseltamivir oral	30
34	49	F	T4	Symmetry	Breast cancer	FCV 700 mg/d 7 days	31
35	68	F	R: T8–9 L: C4	Asymmetry	MM	ACV 750 mg/d 6 days	32
36	30	M	T10	Symmetry	AIDS	?	33
37	64	F	R: L4 L: T10	Asymmetry	PAAS and diabetes	ACV 10 mg/kg tid	34
38	67	F	R: L4–5 L: T7–8	Asymmetry	Hypertension	FCV 750 mg/d 7 days	35
39	69	M	R: T5–7,10,12; L3–4 L: T4–6,12; L3–5	Asymmetry	ESRD and SCCs	ACV IV 800 mg/d 7 days FCV oral 500 mg/d 14 days	36
40	91	F	L2–5	Symmetry	CKD	Antiviral therapy	37

*M* male; *F* female; *L* left; *R* right; *C* cervical; *T* thoracic; *L* lumbar; *S* sacral; *IV* intravenous; *ACV* aciclovir; *FCV* famciclovir; *CLL* chronic lymphocytic leukaemia; *CKD* chronic kidney disease; *ESRD* end-stage renal disease; *MM* multiple myeloma; *AIDS* acquired immune deficiency syndrome; *SLE* systemic lupus erythematosus; *TB* tuberculosis; *UC* ulcerative colitis; *PAAS* polymyositis associated antisynthetase syndrome; *SCCs* multiple squamous cell carcinomas

In the case here, it is curious BHZ and RHS simultaneously happened in an immune-competent patient [5]. In our knowledge, there is no comparable cases have been reported. T cells are critical in the process of VZV delivery, especially for the reactivation of VZV [6]. By reviewing the medical history of this patient, we did not find he experienced any chronic illness nor received any immune suppressant medication. However, it is worth noting that the patient drank in 1 week before the onset of illness as mentioned in some reports that alcohol exposure weakens the body’s defense against virus and even leads to more severe or faster disease progression [7]. Thus, we hypothesize that heavy drinking may be one factor contributing to the reactivation of VZV in the two separate ganglia.

Antivirus therapy is necessary for treating HZ, and early application of glucocorticoid might be useful in reducing swelling and easing inflammation of the nerves [1]. A retrospective study suggested that antivirus - glucocorticoid combination therapy could improve the recovery rate of facial paralysis [8]. Our case confirmed the efficacy of the combination. After admission, the patient received penciclovir 250 mg twice by dripping and methylprednisolone 40 mg once daily immediately. The vesicles in the body had been absorbed, and the pain was alleviated. He also received acupuncture therapy after discharged from the hospital. The facial nerve function of this patient had improved gradually in the months’ follow-up.

## Conclusions

In conclusion, effective antivirus treatment is the key to treat HZ. And antivirus - glucocorticoid combination therapy is necessary for patients who with RHS. Acupuncture therapy may be helpful to the reparation of injury nerves in advanced stages. However, more research is needed to confirm its effectiveness and security.

## Supplementary information

**Additional file 1.** Supplement 1. References for 40 cases in the Table 1.

## Data Availability

Not applicable.

## Abbreviations

AIDS: Acquired immune deficiency syndrome
BHZ: Bilateral herpes zoster
HZ: Herpes zoster
RHS: Ramsay Hunt syndrome
VZV: Varicella-zoster virus
