# Supplementary material for: Bilateral asymmetrical herpes-zoster with Ramsay hunt syndrome in an immunocompetent adult
Source: Virol J. 2020 Aug 15;17:123. doi: 10.1186/s12985-020-01392-0 (PMC7429785; doi:10.1186/s12985-020-01392-0)
Supplement: Supplementary file 1 — Additional file 1. Supplement 1. References for 40 cases in the Table 1. [file 12985_2020_1392_MOESM1_ESM.docx]

Supplement 1

References for 40 cases in the Table：

1. Yumiko T, Yoshiki M, Yoshiaki Y, Miki T, Akihiro F, Yuichiro E. Bilateral disseminated herpes zoster in an immunocompetent host. Dermatology Online Journal. 2013;19:13.

2. Brar BK, Gupta RR, Saghni SS. Bilateral herpes--Zoster of widely separated dermatomes in a non-immunocompromised female. Indian J Dermatol Venereol Leprol. 2002;68:48-9.

3. Pedrosa A, Cruz MJ, Mota A, Baudrier T, Azevedo F. Herpes zoster multiplex and bilateral in an immunocompetent child. PEDIATR INFECT DIS J. 2015;34:225-6.

4. Bloss G, Ebisch MA, Kunz M, Gross G. [Bilateral asymmetric herpes zoster in adolescence]. HAUTARZT. 2001;52:335-8.

5. Gahalaut P, Chauhan S. Herpes zoster duplex bilateralis in an immunocompetent host. Indian Dermatology Online Journal. 2012;3:31-3.

6. Kantaria SM. Bilateral asymmetrical herpes zoster. Indian Dermatol Online J. 2015;6:236.

7. Csontos Z, Sebok B, Karg E, Schneider I. [Bilateral, asymmetric herpes zoster (herpes zoster duplex asymmetricus)]. HAUTARZT. 2001;52:817-9.

8. Nath R. Bilateral and multifocal generalized eruptions in herpes zoster ophthalmicus: case report. INDIAN J OPHTHALMOL. 1996;44:40-2.

9. Finny JM. A Case of Double, or Bilateral, Herpes Zoster. Br Med J. 1885;1:67-8.

10. Vijay A, Dalela G. Herpes Zoster Duplex Bilateralis in Immuno-Competent Patients: Report of Two Cases. J Clin Diagn Res. 2015;9:R1-3.

11. Chen Y, Laguna BA, Marlowe LE, Keller MD, Treat JR. Herpes zoster duplex bilateralis in an immunocompetent adolescent boy: a case report and literature review. PEDIATR DERMATOL. 2014;31:341-4.

12. HAILEY H. Bilateral herpes zoster; report of three cases. SOUTH MED J. 1954;47:728-32.

13. Leung AK, Barankin B. Bilateral symmetrical herpes zoster in an immunocompetent 15-year-old adolescent boy. Case Reports in Pediatrics. 2015;2015:121549.

14. Camb EWPT. BILATERAL ZOSTER : REPORT OF A CASE. LANCET. 1947;250:910-1.

15. Arfan-ul-Bari, Iftikhar N, Ber RS. Bilateral symmetrical herpes zoster in an immunocompetent patient (Herpes zoster duplex symmetricus). J Coll Physicians Surg Pak. 2003;13:524-5.

16. Douglas CE. A Case of Bilateral Herpes Zoster of the Fifth Pair. Br Med J. 1895;1:808-9.

17. Yuval K, Natan G. Delayed oculomotor nerve palsy after bilateral cervical zoster in an immunocompetent patient. NEUROLOGY. 2005;65:170.

18. Lee H, Jung HJ, Park MY, Ann JY. Herpes zoster duplex symmetricus in a healthy patient. Korean Journal of Dermatology. 2011;49:1098-101.

19. Pervez H, Potti A, Mehdi SA. Concomitant bilateral herpes zoster opthalmicus. LANCET INFECT DIS. 2002;2:699.

20. Castronovo C, Nikkels AF. Chronic herpes zoster duplex bilateralis. Acta Derm Venereol. 2012;92:148-51.

21. di Meo N, Bergamo S, Dondas A, Trevisan G. Bortezomib and bilateral herpes zoster. Acta Dermatovenerol Alp Pannonica Adriat. 2012;21:21-2.

22. Brandon EL, Akers J, Rapeport D. Development of bilateral herpes zoster following thoracoscopic splanchnicectomy. Anaesth Intensive Care. 2006;34:382-3.

23. Yau TH, Butrus SI. Presumed bilateral herpes zoster ophthalmicus in an AIDS patient: a case report. CORNEA. 1996;15:633-4.

24. Mobley CA. A CASE OF ASYMMETRICAL, BILATERAL HERPES ZOSTER. Journal of the American Medical Association. 1912;lix:879.

25. Akimoto T, Muto S, Nagata D. Bilateral herpes zoster in a patient with end-stage kidney disease. Int Med Case Rep J. 2017;10:209-12.

26. Ko DH, Park SY, Yoon TJ. A case of bilateral recurrent herpes zoster in multiple myeloma. Korean Journal of Dermatology. 2006;44:1460-3.

27. Lewis RJ, Mitchell JC. Systemic lupus erythematosus, miliary tuberculosis, and bilateral herpes zoster occurring in a Chinese woman. Arch Dermatol. 1971;104:562.

28. Shin BS, Seo HD, Na CH, Choi KC. Case of herpes zoster duplex bilateralis. J DERMATOL. 2009;36:95-7.

29. Gouveia AI, Borges-Costa J, Soares-Almeida L, Santana A, Guerra J. Atypical Presentation of Herpes Zoster Duplex Bilateralis in a Renal Transplanted Patient. Healthcare. 2013;2:20-6.

30. Matsubara H, Konishi T, Saito K, Naito A, Hashizume H. Herpes zoster duplex in a patient with influenza A and bacterial superinfection. The Journal of Dermatology. 2019;

31. Yoo KH, Park JH, Kim BJ, Kim MN, Song KY. Herpes zoster duplex bilateralis in a patient with breast cancer. Cancer Research & Treatment. 2009;41:50-2.

32. Onaka T, Yonezawa A. Herpes zoster duplex bilateralis in bortezomib-based chemotherapy. Blood Res. 2016;51:155.

33. Rajashekar TS, Singh G, Shivakumar V, Okade R. Recurrent herpes zoster duplex symmetricus in HIV infection. INDIAN J DERMATOL. 2008;53:33-4.

34. Peretz A, Nowatzky J, Steiner I. Herpes zoster duplex bilateralis. BMJ Case Rep. 2009;2009:r2006114116.

35. Shin JW, Kim DH, Whang KU, Lee J, Park Y, Cho MK, Lee S, Lee SY. A case of zoster duplex bilateralis. ANN DERMATOL. 2009;21:423-5.

36. Vu AQ, Radonich MA, Heald PW. Herpes zoster in seven disparate dermatomes (zoster multiplex): report of a case and review of the literature. J AM ACAD DERMATOL. 1999;40:868-9.

37. Puntillo F, Giglio M, Preziosa A, Mele R, Brienza N. A Bilateral Lumbar Multidermatomal Herpes Zoster in an Elderly Woman with Chronic Kidney Disease. Pain and therapy. 2020;
